# Evaluation of Physical Properties of a Metakaolin-Based Alkali-Activated Binder Containing Waste Foam Glass

**DOI:** 10.3390/ma13235458

**Published:** 2020-11-30

**Authors:** Petra Mácová, Konstantinos Sotiriadis, Zuzana Slížková, Petr Šašek, Michal Řehoř, Jaroslav Závada

**Affiliations:** 1 Institute of Theoretical and Applied Mechanics of the Czech Academy of Sciences, Prosecká 809/76, 19000 Prague, Czech Republic; macova@itam.cas.cz (P.M.); slizkova@itam.cas.cz (Z.S.); 2 Department of Building Materials and Products, South Ural State University (National Research University), pr. Lenina 76, 454080 Chelyabinsk, Russia; 3 Continental Automotive Czech Republic s.r.o., Hradecká 1092, 50601 Jičín, Czech Republic; petr.sasek@continental-corporation.com; 4 Brown Coal Research Institute JSC, Budovatelů 2830/3, 43401 Most, Czech Republic; rehor@vuhu.cz; 5 Albrechtova Střední Škola, Tyršova 611/2, 73701 Český Těšín, Czech Republic; jaroslav.zavada@albrechtovastredni.cz; 6 Faculty of Mining and Geology, VŠB—Technical University of Ostrava, 17. listopadu 2172/15, 70800 Ostrava-Poruba, Czech Republic

**Keywords:** alkali-activated materials, metakaolin, foam glass, waste, composite materials, thermal insulation

## Abstract

Foam glass production process redounds to large quantities of waste that, if not recycled, are stockpiled in the environment. In this work, increasing amounts of waste foam glass were used to produce metakaolin-based alkali-activated composites. Phase composition and morphology were investigated by means of X-ray powder diffraction, Fourier-transform infrared spectroscopy and scanning electron microscopy. Subsequently, the physical properties of the materials (density, porosity, thermal conductivity and mechanical strength) were determined. The analysis showed that waste foam glass functioned as an aggregate, introducing irregular voids in the matrix. The obtained composites were largely porous (>45%), with a thermal conductivity coefficient similar to that of timber (<0.2 W/m∙K). Optimum compressive strength was achieved for 10% incorporation of the waste by weight in the binder. The resulting mechanical properties suggest the suitability of the produced materials for use in thermal insulating applications where high load-bearing capacities are not required. Mechanical or chemical treatment of the waste is recommended for further exploitation of its potential in participating in the alkali activation process.

## 1. Introduction

Foam glass is a thermal-insulating and soundproof material with a history dating back to 1930s, when the pioneering developments were realized in France and the former Soviet Union. Later on, several studies were performed in this field in the United States, United kingdom, Germany, Czech Republic and Japan [1]. It is produced by mixing fine glass powder of 100–500 μm (nowadays, almost entirely supplied as ground recycled glass) with a foaming agent of 75–150 μm (e.g., CaSO_4_, CaCO_3_, water glass, aluminum slag, coal and SiC) and firing the mixture at 700–900 °C. At such temperatures, the glass melts while the foaming agent decomposes, resulting in the formation, upon cooling, of a rigid cellular material [2] with porosity and pore diameter of 80–90% and 0.1–5 mm, respectively [3]. The technical characteristics of foam glass (thermal conductivity coefficient in the range 0.03–0.05 W/m∙K, satisfactory strength of 1 MPa, moisture impermeable and fire-resistant) make it attractive for several thermal insulation applications [4]. Three product types of foam glass are distinguished: blocks, granules (pellets) and glass aggregate (gravel). The latter is obtained by crushing the waste trimmed during the production of blocks [2,4].

Because of the attractive properties of foam glass, the waste can be utilized, alternatively, for the production of materials appropriate for building applications. The combination with a binder of low environmental impact that will serve as the matrix hosting the waste can be considered for the design of sustainable composites. In this aspect, alkali-activated materials can be viewed as a favorable choice, since either an industrial by-product (e.g., fly ash, blast furnace slag, red mad and sludge) or metakaolin (MK, processed from kaolin clay by calcination at moderate temperatures 650–800 °C) can be used as the aluminosilicate source [5,6,7], along with an easily produced alkaline activator (concentrated aqueous solution of alkali hydroxide, silicate, carbonate or sulfate) [8]. The alkaline conditions cause the destruction of the Si-O-Si, Si-O-Al and Al-O-Al covalent bonds, bringing the constituent tetrahedra of the raw material to a discrete state. This process is catalyzed by the alkali metal cations (Na^+^, K^+^ and Li^+^), later participating together with alkaline earth metal cations (Ca^2+^, Mg^2+^), possibly present in the system, in the formation of complex hydrates [9], whose nature depends on the aluminosilicate precursor [10]. Waste foam glass (WFG) may dissolve in the prevailing alkaline conditions [11] and, thus, participate in the geopolymerization process.

Several studies [11,12,13,14,15,16,17,18,19,20,21,22,23] have investigated the utilization of waste glass in the production of alkali-activated materials, in combination with different aluminosilicate raw materials (metakaolin, fly ash and blast furnace slag). Common sources of waste glass included foam glass, glass cullets, solar panel glass, old glass bottles, municipal waste glass and fluorescent lamps at the end of their service life. In these studies, waste glass either played the role of aggregate/filler [12,13,14,15,16,17] or functioned as a precursor for geopolymerization [11,14,15,16,18,19,20,21], while it was also employed for the preparation of the alkali activators [22,23]. Previous studies have shown that the incorporation of increasing amounts of waste glass in alkali-activated materials caused a decrease in the mechanical properties, attributed to the increase in Si/Al ratio achieved by its use [11], solid-to-liquid ratio [13], mean particle size [15] and micro-cracks formed in the matrix [12]. Furthermore, decrease in density and increase in porosity have been also observed [13,17]. Fine waste glass powders were found to participate in the geopolymerization process, contributing in strength increase [16,20,21,24].

The objective of our work was to investigate the effect of WFG incorporation in a metakaolin-based alkali-activated binder on its physical properties (density, porosity, strength and thermal conductivity) and microstructure. The overall characteristics of the produced materials may indicate the field of their application, as well as any pretreatment path of WFG for modifying the materials’ properties. The research significance draws from exploring the possibility to recycle the large quantities of WFG generated during the industrial-scale manufacture of foam-glass insulating products, for the production of lightweight environmentally friendly materials for thermal-insulating applications.

## 2. Materials and Methods

Commercial metakaolin, MK, (Mefisto K05, ČLUZ a.s., Czech Republic) and waste foam glass, WFG, (Pittsburgh Corning CR s.r.o., Klášterec nad Ohří, Czech Republic) were used as solid components for the preparation of alkali-activated mixtures. The chemical composition of raw materials is reported in Table 1, as derived from silicate analysis performed according to Reference [25].

**Figure 1:**
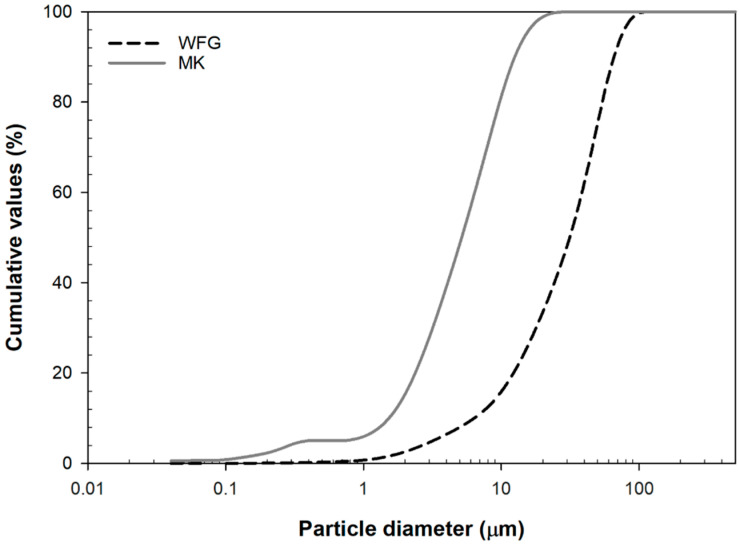
Cumulative particle-size distribution curves of the raw materials (MK and WFG).

Cumulative particle-size distribution curves of the MK and WFG, as determined with a CILAS 1090 particle size analyzer, are provided in Figure 1. Mean particle size (*d*_50_) of 5.2 and 31.4 μm, and BET surface area of 13.98 and 0.59 m^2^/g, were determined for MK and WFG, respectively. The latter was figured out according to the standard ISO 9277:2010(E) [26], using a Micrometrics ASAP 2020 instrument, by calculating the Langmuir surface area based on the volume of gas adsorbed by the meso- and macropores. Sodium water-glass (Kittfort s.r.o., Neratovice, Czech Republic) of silicate modulus M_s_ = 3.78, determined according to Reference [27], was employed as the liquid component (alkaline activator). The adjustment of the silicate modulus to 1.30 was performed by using a saturated NaOH solution, produced from reagent-grade NaOH (Lach-Ner, s.r.o., Neratovice, Czech Republic). The SiO_2_, Na_2_O and H_2_O contents in water glass were 28.96%, 7.90% and 63.13%, respectively.

Four mixtures were produced by WFG substitution for MK by 10%, 30% and 50% by mass, according to the compositions of Table 2. The “total H_2_O/(MK+WFG)” and “Na_2_O/(MK+WFG)” ratios were equal to 0.89 and 0.15, respectively. The term “total H_2_O” equals 288 g and corresponds to the sum of the water added in the mixture (147.60 g), the water included in the water glass used (131.28 g) and the water derived from NaOH (9.12 g). The total amount of solids (432 g) consists of the raw materials (324 g), Na_2_O (47.78 g) and SiO_2_ (60.22 g). Thus, the proportion of water in each mixture was 40%.

Each mixture was initially hand-mixed for 2 min, and then mechanically mixed for 5 min, with a Wisd WiseStir HT-120DX shaft mixer (WITEG Labortechnik GmbH, Wertheim, Germany), at 350 rpm, and finally homogenized for 2 min, in an ultrasound bath. The produced mixtures were poured in prismatic moulds (20 × 20 × 100 mm) of appropriate capacity to prepare six specimens per each mixture and stored in a climatic chamber, at controlled conditions (40 ± 1 °C; 60 ± 5% relative humidity), for 24 h, while being sealed with polypropylene foil. After demolding, the specimens were further stored in a climatic chamber (20 ± 1 °C; 65 ± 5% RH) for 27 days.

The mineralogical and morphological characterizations of the raw materials and the produced alkali-activated materials were performed with X-ray powder diffraction (XRPD), Fourier-transform infrared (FTIR) spectroscopy and scanning electron microscopy (SEM). For the former two analytical methods, powder samples (one for each composition) were prepared by hand-grinding the relevant materials into an agate mortar.

XRPD data were collected in the angular range 5–82° 2*θ*, using a Bragg–Brentano *θ*−*θ* diffractometer (Bruker D8 Advance), Cu radiation (Kα = 1.5406 Å), at 40 kV and 40 mA, applying a virtual step scan of 0.01° 2*θ* with 0.4 s/step counting time. The side loading technique was used to minimize, a priori, the preferred orientation of crystallites. The samples were spun at 15 rpm, to improve particle statistics. The evaluation of data was accomplished with the X’Pert HighScore Plus 2.0 software (Malvern Panalytical Ltd., Malvern, UK), by employing the ICDD PDF-2 database.

FTIR measurements were carried out with a secondary module iZ10 of Nicolet iN10 FTIR microscope (Thermo Scientific, USA) equipped with an attenuated total reflectance (ATR) (diamond) crystal. Spectra were collected by an accumulation of 64 scans, at 4 cm^−1^ resolution, in the spectral range of 4000–525 cm^−1^.

SEM observations were performed at 20 kV accelerating voltage and vacuum conditions, employing a FEI QUANTA FEG 450 instrument (Thermo Fischer Scientific, Waltham, MA, USA) equipped with a Schottky type electron source, an Everhart-Thornley detector in secondary electron mode, and an energy dispersive (EDS) detector. Prior to analysis the samples were mounted on aluminum stubs and then coated with a 5 nm thick gold film, using a Quorum Q150 R instrument (Quorum Technologies Ltd., Lewes, UK).

After the 28-day initial curing, the density of the produced specimens was calculated from mass and volume measurements on six specimens per composition. Flexural and compressive strength were measured at 28 days after specimens’ preparation, on six and three specimens for each composition, respectively, using an MTS Criterion C45 electromechanical test system (MTS Systems Corporation, Eden Prairie, MN, USA) fitted with a 5 kN load cell, applying a loading rate of 0.5 mm/min. Mercury intrusion porosimetry (MIP) was used to determine the total porosity and the pore-size distribution of the hardened specimens at 28 days (two samples per composition), employing a Micrometrics AutoPore IV 9500 porosimeter (Micrometrics Instrument Corp., Norcross, GA, USA), at a maximum pressure of 227.5 MPa. The covered range in pore diameter was 0.005–375 μm. Heat-transfer properties were assessed by measuring thermal conductivity coefficient (*λ*) on two specimens per composition, engaging an ISOMET 2104 apparatus (Applied Precision Ltd., Rača, Slovakia). The results presented in the next sections are the average values of the measured quantities.

## 3. Results

### 3.1. Phase Characterization

**Figure 2:**
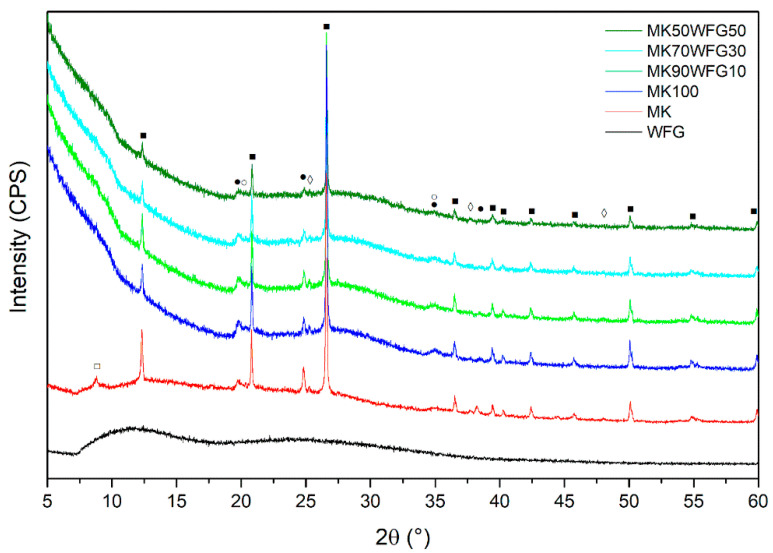
XRPD patterns of WFG, MK and of the produced alkali-activated materials (MK100, MK90WFG10, MK70WFG30 and MK50WFG50). The main peaks are indicated as follows: ▪—quartz; ●—kaolinite; □—muscovite; ◊—anatase.

XRPD patterns of the WFG, MK and of the produced composites are presented in Figure 2. XRPD analysis indicated the mainly amorphous nature of MK, consisting also from a number of crystalline phases, namely quartz, anatase, kaolinite and muscovite. Anatase is due to the presence of TiO_2_ in MK (Table 1); the latter two phases are clay relics which had not been decomposed during the thermal activation of the original kaolinitic clay [7]. WFG was found to be totally amorphous, as expected [11,12,20,28]. The XRPD pattern of the MK100 sample points to a largely amorphous material containing quartz and kaolinite inclusions, consistent with previous results on metakaolin-based alkali-activated binders [7,29], as well as traces of anatase. This phase assemblage was maintained in the composite specimens (MK90FG10, MK70FG30 and MK50FG50). The lower intensities observed, mainly, for the kaolinite peaks, are attributed to the lower amount of MK used in the mixtures with WFG. The main difference between the raw MK and the produced alkali-activated materials is the consumption of muscovite. Although WFG is rich in sodium, Na-containing crystalline phases were not detected with XRPD; thus, any possible dissolution of elements from WFG by the alkaline conditions led to their inclusion in the amorphous phase.

**Figure 3:**
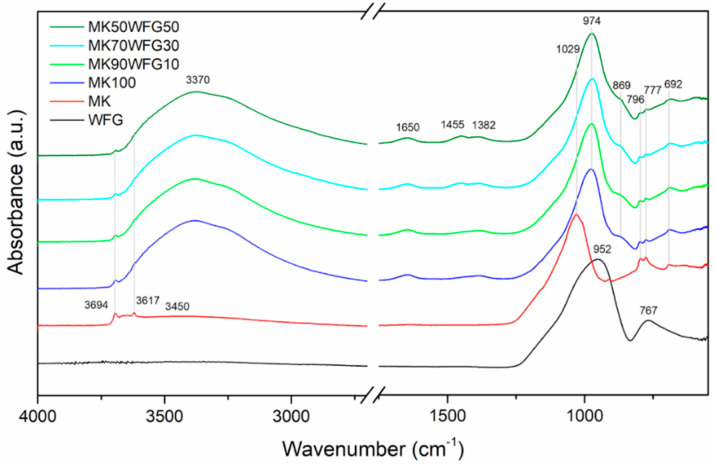
FTIR spectra of WFG, MK and of the produced alkali-activated materials (MK100, MK90WFG10, MK70WFG30 and MK50WFG50). The wavenumbers of the main bands are indicated. The intensity of the spectra in the range 4000–2700 cm^−1^ was manipulated for illustrative purposes.

FTIR spectra obtained for WFG, MK and for the produced composites are shown in Figure 3. The spectrum of WFG comprises typical bands for a glass [20,22], i.e., asymmetric stretching vibration of Si-O-Si and Si-O-Al bonds (952 cm^−1^), and symmetric stretching vibration of Si-O-Si bonds (767 cm^−1^). The FTIR spectrum of MK indicates a main contribution by the asymmetric stretching vibration of Si-O-Si and Si-O-Al bonds at 1029 cm^−1^ [12,22,30]. Most of the silicon sites are present in a 2D sheet consisting mainly of *Q*^4^(1Al) environments, thus Si-O-Si bonds are considered to contribute the most to this signal [31,32]. In this spectrum, the peak at 796 cm^-1^ can be attributed to Al^(IV)^-O and Si-O bending vibrations [22,33] or to the stretching vibrations of six-coordinated Al^(VI)^-O [34]. The band at 777 cm^−1^ is related to the presence of quartz in MK, similarly to fly ash [11,17]. Stretching vibrations of O-H groups, which appeared in the spectra as sharp peaks at 3694 and 3617 cm^−1^ and as broad band with the maximum at around 3450 cm^−1^, are attributed to chemically bonded hydroxyl groups of kaolinite residue and to physically adsorbed water, respectively [22,24].

The alkali activation and polycondensation processes caused a shift of the major intense band in the spectrum of MK (1029 cm^−1^) by about 55 cm^−1^ towards lower wavenumbers. This is consistent with the findings of previous studies in metakaolin-based geopolymers [12,22,28,30,33,34]. The decrease in wavenumber is indicative of angle and length changes of Si-O-Si and Si-O-Al bonds [28] and attributed to the substitution of SiO_4_ tetrahedra for AlO_4_ tetrahedra in the silicate network and to the occurrence of non-bridging oxygen sites [31,34]. Further, the band at 796 cm^−1^ was not observed in the spectra of the produced alkali-activated materials, in agreement with other studies [22,33], as a result of the change in Al coordination (from 6 to 4) and its participation in the silicate chains [34]. This is also confirmed by the appearance of the band at 692 cm^−1^ [22]. The band at 869 cm^−1^, not observed in MK spectrum, is attributed to the bending vibration of -OH in Si-OH groups [34]. The wide band at around 3370 cm^−1^ and the new one arising at 1650 cm^−1^, assigned to stretching and bending vibrations of O-H groups [15,22,33], respectively, denote the inclusion of water molecules in the geopolymeric frame [30]. Two more bands at 1455 cm^−1^ and 1382 cm^−1^, observed in the FTIR spectra of the alkali-activated materials, are assigned to stretching vibrations of C-O-C bonds in CO_3_^2−^ groups, most likely related to efflorescence on their surface, as a result of the reaction between the unreacted activator (NaOH) and the atmospheric CO_2_ [28,35]:

2NaOH (dissolved) + CO_2_ (dissolved) → Na_2_CO_3_ (solid) + H_2_O (liquid), (1)

These bands were apparently more intense in the case of the specimens MK70WFG30 and MK50WFG50. It is possible that the conditions that prevailed in the fresh mixture favored the partial leaching of Na from WFG that enhanced efflorescence.

### 3.2. Morphological Characterization

**Figure 4:**
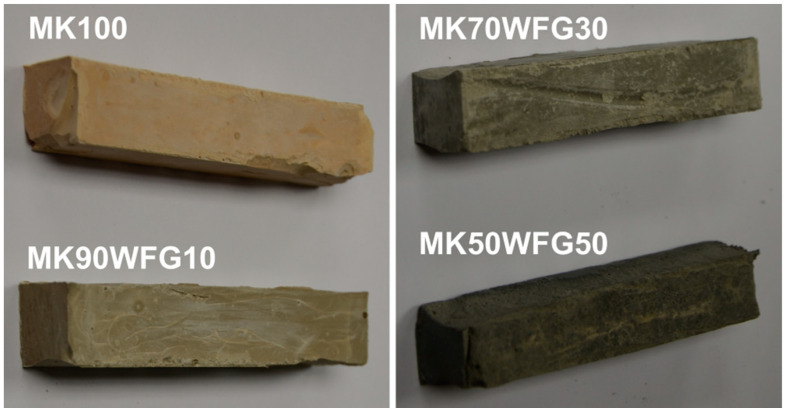
Appearance of the 28-day cured specimens.

Illustrative photos of the appearance of the 28-day-cured specimens are shown in Figure 4; WFG content has a noticeable effect on their color.

**Figure 5:**
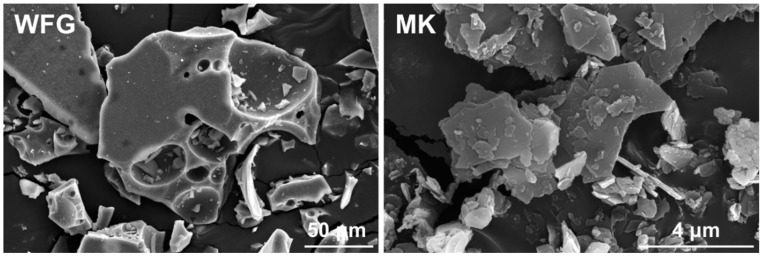
SEM micrographs of the raw materials (WFG and MK).

SEM micrographs of WFG and MK are illustrated in Figure 5. WFG particles are irregular in shape, with sharp edges and a smooth surface, including spherical-shaped voids of different size that are inherited from the entrapment of air during the production process [36]. MK has the typical structure of this material [33], consisting of plate-like aluminosilicate sheets that are piled as distinct clusters.

**Figure 6:**
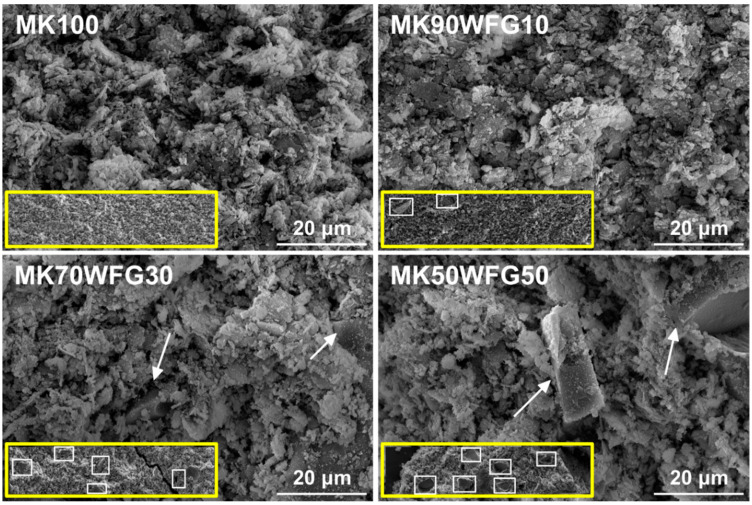
SEM micrographs of the produced alkali-activated materials (MK100, MK90WFG10, MK70WFG30 and MK50WFG50) at 1500× magnification (600× magnification images given as insets; WFG particles in the matrices are indicated).

SEM micrographs of fragments obtained from the hardened specimens of the four compositions are depicted in Figure 6. The micrographs of the samples MK100 and MK90WFG10 are quite similar, indicating that the microstructure of the alkali-activated binder is not significantly affected by the incorporation of small amount (10%) of WFG. The images at lower magnification (600×), shown as insets, illustrate the scale-like homogenous matrix of the binder MK100, typical of this type of material [33], and the scarce presence of WFG particles into the matrix of sample MK90WFG10. For higher substitution levels (30% and 50%), a lack of cohesion between the binder and the WFG particles is noticeable, being responsible for the presence of large interconnected irregular voids. In both samples, well-distributed WFG particles in the matrices are distinguished (see insets).

### 3.3. Physical and Mechanical Properties

**Figure 7:**
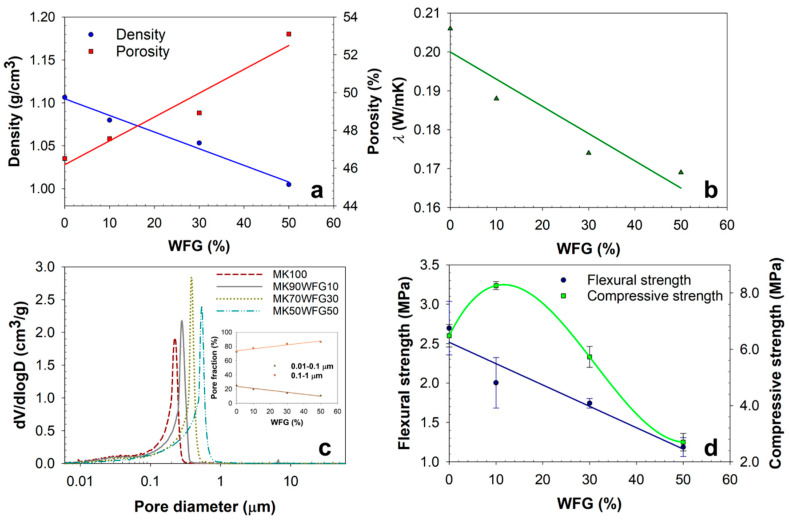
Density and porosity (**a**), thermal conductivity coefficient (**b**), pore─size distribution (**c**) and mechanical strength (**d**) of the produced alkali-activated materials versus the % amount of WFG used.

The physical characteristics of the produced alkali-activated materials, i.e., density, MIP porosity, thermal conductivity coefficient, pore-size distribution and mechanical properties, as a function of the WFG content, are summarized in Table 3 and illustrated in Figure 7. Regression lines, derived from fitting the data to polynomial functions, were used solely for highlighting the observed trends. Details regarding the porosity measurements are reported in Table 4.

Apparently, increasing the WFG content resulted in increasing porosity (Figure 7a). Since only open porosity is accessible with MIP, the mostly closed porosity of WFG particles (Figure 5) did not contribute in the values obtained; thus, it can be implied that the increase observed is most likely due to the increase of voids in the matrix, as detected with SEM. However, a contribution from cracking of WFG under the pressure applied during the experiment cannot be excluded. The increase in porosity directly affected the density (Figure 7a) and thermal conductivity coefficient (Figure 7b), both decreasing with increasing WFG content. It should be noted that there is a very good agreement between the density values determined from mass and volume measurements (Table 3) and MIP measurements (Table 4). The values are consistent with the results of Hao et al. [13], regarding metakaolin-based geopolymers incorporating solar panel waste glass. Further, the increase in WFG content shifted the pore-size distribution towards larger pore sizes (Figure 7c), decreasing at the same time the contribution of smaller pores to the total porosity. The coarsening of the microstructure is also indicated by the increase in the total intrusion volume and average pore diameter, and by the decrease in the total pore area (Table 4). In fact, all curves demonstrate that this is mainly due to the contribution of two pore ranges, i.e., 0.01–0.1 μm and 0.1–1 μm. The diagram depicted as an inset in Figure 7c indicates the significant decrease (up to ~57%) and increase (up to ~20%) of the amount of pores classified in the two pore-size ranges, respectively. Most likely, the reduction of the number of pores in the range 0.01−0.1 μm is due to the decrease of the MK content in the mixtures incorporating increasing amounts of WFG, and thus of the geopolymer gel formed. The porosity of metakaolin-based alkali activated binders is largely contributed by this pore-size range [37].

The structural differences among the materials of the four compositions were reflected on the mechanical properties (Figure 7d). Flexural strength decreased with increasing WFG content almost linearly; reduced values by 25.7% (MK90FG10), 35.4% (MK70FG30) and 56.0% (MK50FG50) were measured, in comparison to the reference binder (MK100). Regarding compressive strength, the lowest WFG replacement level (10%) led to a 27.5% increase of strength value; however, further increase in WFG content decreased the compressive strength by 11.6% (MK70FG30) and 58.4% (MK50FG50), compared to MK100. The overall low strength values obtained can be linked to the high proportion of water (40%) in the mixtures. A previous study on metakaolin-based geopolymers showed that such water proportion resulted in compressive and flexural strength values analogue to the findings of the current work [38]. Regardless of WFG use in the mixtures, the mechanical properties of the pure binder (MK100) were also quite low, indicating that metakaolin was likely not effectively dissolved by the applied alkaline conditions, thus hampering gel formation and the subsequent strength development. Chen et al. [39] found that the employment of Na_2_O-to-metakaolin ratios in the range of 0.42–0.61 (compare to 0.15 used in our work) resulted in compressive strength values of the metakaolin binder by 4.1–5.8 times higher than the currently obtained ones (Table 3 and Figure 7). Further, the applied curing conditions (approximately 60% RH) enhanced the evaporation of water, hindering also the geopolymerization process.

## 4. Discussion

All experimental results suggest that WFG dissolved to a limited extent and, thus, functioned mostly as an aggregate. This can be attributed to its coarser nature, compared to MK; only a 20% fraction of WFG particles have a size similar to that of MK (<10 μm) that may have contributed to geopolymerization. The incorporation of increasing amounts of WFG, accompanied by a corresponding decrease of the amount of MK, without any change in the liquid component (alkali activating solution (water glass, NaOH solution) and added water) of the mixtures, resulted in the introduction of voids in the composite material with a size mainly in the range of 0.1−1 μm. The amount of smaller pores (0.01−0.1 μm) decreased instead. As observed in SEM micrographs, most of the formed pores were irregular in shape. The decrease in the ratio of reactive component (less MK, more WFG) caused the presence of excess water and NaOH in the mixtures. According to Barbosa et al. [40], excess water induces leaching of the more soluble component, which, in our case, is MK, because of its specific surface (MK: 13.98 m^2^/g; WFG: 0.59 m^2^/g). Thus, less gel formation is expected, affecting the consistency of the fresh paste and, subsequently, the homogeneity of the resulting composites. Further, voids may form in the matrix as a result of partial evaporation of the water in excess, during the thermal curing of the specimens. The excess NaOH in the mixtures containing WFG caused efflorescence, as detected in the FTIR spectra and suggested in Reference [40], which increased with the WFG content.

The structural changes induced by WFG particles had a remarkable effect on density and porosity, and, consequently, on thermal conductivity and mechanical strength, compared to the reference specimen (MK100). In fact, *λ* was measured below 0.2 W/m∙K independently of the WFG substitution level of the binder. Such *λ* values are similar to those of timber [41]. Regarding mechanical properties, they dropped as a result of the brittle nature of WFG and the voids created in the matrices, due to the reasons mentioned previously. Nevertheless, low amounts of WFG (10% w/w) can be properly hosted by the alkali-activated binder matrix, developing bonds that likely counteract the brittle nature of the waste and enhance the compressive strength, compared to the pure binder (MK100). Correspondingly to the present findings, previous studies [12,15] showed a drop in compressive strength of alkali-activated metakaolin or fly ash binders containing waste glass, attributed to the micro-cracks introduced in the matrix as a result of the extensive presence of unreacted waste glass. Further, the improving effect of waste glass incorporation, up to a certain amount, on compressive strength, was also observed before [12,14]. A decrease of the liquid component may be recommended to increase the mechanical properties of the produced composites; however, the mixture has to be carefully designed in order to avoid any significant deteriorating effect on the thermal properties. Other factors to be considered include the curing at higher temperature and humidity conditions that may facilitate the development of mechanical properties [42] and prevent the evaporation of water, respectively, as well as the use of an activator of higher alkalinity to enhance MK dissolution.

The overall characteristics of the obtained composites (large porosity, low thermal conductivity coefficient and low mechanical strength) suggest their use as thermal insulating materials in applications not experiencing intense mechanical or thermal loading that could impair their structural integrity and, thus, their functionality. For comparison, typical values of thermal conductivity coefficient and compressive strength are in the range of 0.030–0.230 W/m∙K and 0.04–1.5 MPa, respectively, for common thermal insulating materials [4]. The obtained composites are considered to have satisfactory properties, given that the purpose of the study was to exploit the waste as supplied by the factory, without applying any pretreatment, for eliminating any further environmental and economic impact. Nevertheless, considering the chemical composition of WFG, its potential to function as a precursor for the production of alkali-activated materials, complementary to MK, can be considerably improved by either increasing its fineness and employing higher alkalinity conditions [15,24] or by following a pre-activation procedure [23] for facilitating its dissolution and the subsequent polycondensation reactions. Since the dissolution of WFG is affected by the intensity of the alkaline conditions [12] and the curing temperature of the fresh mixtures [43], both parameters have to be taken into consideration for the mix design.

## 5. Conclusions

The physical properties of metakaolin-based alkali-activated composites incorporating increasing amounts of waste foam glass were assessed in view of the phase composition and morphology. The following conclusions can be drawn from the investigation.

The obtained materials are characterized by large porosity (always >45%) and a low thermal conductivity coefficient (always <0.2 W/m∙K). Their relatively low mechanical properties indicate the suitability for thermal-insulation applications that do not demand materials of high load-bearing capacity. The overall characteristics of the produced composites suggest their use as prefabricated elements attached appropriately on building surfaces. The incorporation of 10% waste foam glass by weight in the binder was found to provide the optimum compressive strength, while retaining low thermal conductivity. The results suggest that waste foam glass can be appropriately recycled in the production of thermal insulating materials as it is, without any prior pretreatment, thus avoiding any further environmental or economic impact. The waste foam glass played the role of aggregate; however, particle-size reduction and chemical activation processes are recommended for the exploitation of its overall potential.

## Figures and Tables

**Table 1:** Chemical composition (wt.%) of the raw materials (metakaolin (MK) and waste foam glass (WFG)).

Raw Material	SiO_2_	Al_2_O_3_	Fe_2_O_3_	CaO	MgO	MnO	Na_2_O	K_2_O	P_2_O_5_	TiO_2_	SO_3_	LOI	Humidity
MK	56.90	38.12	0.72	0.72	0.30	0.01	0.19	0.67	0.05	0.42	0.02	1.80	0.50
WFG	64.03	7.37	3.99	4.76	1.99	0.42	14.89	1.45	0.32	0.61	0.17	0.90	0.30

**Table 2:** Alkali-activated mixtures’ compositions.

Code	MK (g)	WFG (g)	Water Glass (g)	NaOH (g)	H_2_O (g)
MK100	324.0	0	207.93	40.48	147.60
MK90WFG10	291.6	32.4	207.93	40.48	147.60
MK70WFG30	226.8	97.2	207.93	40.48	147.60
MK50WFG50	162.0	162.0	207.93	40.48	147.60

**Table 3:** Summary of the values of physical and mechanical properties of the produced composites.

WFG (%)	Density (g/cm^3^)	Porosity (%)	*λ* (W/m·K)	Flexural Strength (MPa)	Compressive Strength (MPa)
0	1.11	46.5	0.206	2.70	6.48
10	1.08	47.6	0.188	2.00	8.27
30	1.05	48.9	0.174	1.74	5.73
50	1.01	53.1	0.169	1.19	2.70

**Table 4:** Average values of mercury intrusion porosimetry (MIP) parameters determined for the produced composites.

WFG (%)	Total Intrusion Volume (mL/g)	Total Pore Area (m^2^/g)	Average Pore Diameter (Å)	Bulk Density At 0.53 Psia (g/mL)
0	0.42	19.8	1881	1.13
10	0.43	17.6	2479	1.09
30	0.47	13.8	3484	1.05
50	0.51	11.4	4372	1.04

## References

[1] Cui S.P., Zhang J.G., Tian Y.L., Sun S.B., Wu Z.W., Liu W.C. (2014). Generation review on the production line development of foam glass at home and abroad. Adv. Mater. Res..

[2] El-Haggar S.M. (2007). Sustainable Industrial Design and Waste Management.

[3] Zhang H. (2011). Building Materials in Civil Engineering.

[4] Manevich V.E., Subbotin K.Y. (2008). Foam glass and problems of energy conservation. Glas. Ceram..

[5] Ferone C., Capasso I., Bonati A., Roviello G., Montagnaro F., Santoro L., Turco R., Cioffi R. (2019). Sustainable management of water potabilization sludge by means of geopolymers production. J. Clean. Prod..

[6] Yang Z., Mocadlo R., Zhao M., Sisson R.D., Tao M., Liang J. (2019). Preparation of a geopolymer from red mud slurry and class F fly ash and its behavior at elevated temperatures. Constr. Build. Mater..

[7] Sotiriadis K., Guzii S.G., Mácová P., Viani A., Dvořák K., Drdácký M. (2019). Thermal Behavior of an Intumescent Alkaline Aluminosilicate Composite Material for Fire Protection of Structural Elements. J. Mater. Civ. Eng..

[8] Provis J.L. (2014). Geopolymers and other alkali activated materials: Why, how, and what?. Mater. Struct. Constr..

[9] Krivenko P. (2017). Why alkaline activation—60 years of the theory and practice of alkali-activated materials. J. Ceram. Sci. Technol..

[10] Li C., Sun H., Li L. (2010). A review: The comparison between alkali-activated slag (Si + Ca) and metakaolin (Si + Al) cements. Cem. Concr. Res..

[11] Bobirică C., Shim J.H., Pyeon J.H., Park J.Y. (2015). Influence of waste glass on the microstructure and strength of inorganic polymers. Ceram. Int..

[12] Chokkha S., Phetnat P., Chandadi W., Srisitthigul M. (2017). Use of waste glass as a reinforce material in calcined-kaolin based geopolymer. Key Eng. Mater..

[13] Hao H., Lin K.-L., Wang D., Chao S.-J., Shiu H.-S., Cheng T.-W., Hwang C.-L. (2013). Utilization of solar panel waste glass for metakaolinite-based geopolymer synthesis. Environ. Prog. Sustain. Energy.

[14] Novais R.M., Ascensão G., Seabra M.P., Labrincha J.A. (2016). Waste glass from end-of-life fluorescent lamps as raw material in geopolymers. Waste Manag..

[15] Toniolo N., Taveri G., Hurle K., Roether J.A., Ercole P., Dlouhý I., Boccaccini A.R. (2017). Fly-Ash-Based geopolymers: How the sddition of recycled glass or red mud waste influences the structural and mechanical properties. J. Ceram. Sci. Technol..

[16] Xiao R., Ma Y., Jiang X., Zhang M., Zhang Y., Wang Y., Huang B., He Q. (2020). Strength, microstructure, efflorescence behavior and environmental impacts of waste glass geopolymers cured at ambient temperature. J. Clean. Prod..

[17] Kristály F., Szabó R., Mádai F., Debreczeni Á., Mucsi G. (2020). Lightweight composite from fly ash geopolymer and glass foam. J. Sustain. Cem. Mater..

[18] Cyr M., Idir R., Poinot T. (2012). Properties of inorganic polymer (geopolymer) mortars made of glass cullet. J. Mater. Sci..

[19] Rashidian-Dezfouli H., Rangaraju P.R. (2017). Comparison of strength and durability characteristics of a geopolymer produced from fly ash, ground glass fiber and glass powder. Mater. Constr..

[20] Tho-In T., Sata V., Boonserm K., Chindaprasirt P. (2016). Compressive strength and microstructure analysis of geopolymer paste using waste glass powder and fly ash. J. Clean. Prod..

[21] Zhang S., Keulen A., Arbi K., Ye G. (2017). Waste glass as partial mineral precursor in alkali-activated slag/fly ash system. Cem. Concr. Res..

[22] El-Naggar M.R., El-Dessouky M.I. (2017). Re-use of waste glass in improving properties of metakaolin-based geopolymers: Mechanical and microstructure examinations. Constr. Build. Mater..

[23] Torres-Carrasco M., Puertas F. (2015). Waste glass in the geopolymer preparation. Mechanical and microstructural characterisation. J. Clean. Prod..

[24] Toniolo N., Rincón A., Roether J.A., Ercole P., Bernardo E., Boccaccini A.R. (2018). Extensive reuse of soda-lime waste glass in fly ash-based geopolymers. Constr. Build. Mater..

[25] (2009). ÚNMZ Basic Analysis of Silicates—Common Regulations, ČSN 72 0100 2009.

[26] ISO [International Organization for Standardization] (2010). Determination of the Specific Surface Area of Solids by Gas Adsorption—BET Method.

[27] Bednařík V., Vondruška M. (2008). Conductometric analysis of water glass. Chem. List..

[28] Bai C., Li H., Bernardo E., Colombo P. (2019). Waste-to-resource preparation of glass-containing foams from geopolymers. Ceram. Int..

[29] Sotiriadis K., Guzii S., Kumpová I., Mácová P., Viani A. (2017). The effect of firing temperature on the composition and microstructure of a geocement-based binder of sodium water-glass. Solid State Phenom..

[30] Tchakoute Kouamo H., Elimbi A., Mbey J.A., Ngally Sabouang C.J., Njopwouo D. (2012). The effect of adding alumina-oxide to metakaolin and volcanic ash on geopolymer products: A comparative study. Constr. Build. Mater..

[31] Provis J.L., Yong S.L., Van Deventer J.S.J., Scrivener K., Favier A. (2015). Characterising the reaction of metakaolin in an alkaline environment by XPS, and time- and spatially-resolved FTIR spectroscopy. Calcined Clays for Sustainable Concrete.

[32] White C.E., Provis J.L., Proffen T., Riley D.P., Van Deventer J.S.J. (2010). Combining density functional theory (DFT) and pair distribution function (PDF) analysis to solve the structure of metastable materials: The case of metakaolin. Phys. Chem. Chem. Phys..

[33] Kljajević L.M., Nenadović S.S., Nenadović M.T., Bundaleski N.K., Todorović B., Pavlović V.B., Rakočević Z.L. (2017). Structural and chemical properties of thermally treated geopolymer samples. Ceram. Int..

[34] Yunsheng Z., Wei S., Zongjin L. (2010). Composition design and microstructural characterization of calcined kaolin-based geopolymer cement. Appl. Clay Sci..

[35] Allahverdi A., Najafi Kani E., Hossain K.M.A., Lachemi M., Pacheco-Torgal F., Labrincha J.A., Leonelli C., Palomo A., Chindaprasirt P. (2015). Methods to control efflorescence in alkali-activated cement-based materials. Handbook of Alkali-Activated Cements, Mortars and Concretes.

[36] Gong X.Z., Tian Y.L., Zhang L.J. (2018). A comparative life cycle assessment of typical foam glass production. Mater. Sci. Forum.

[37] Yan D., Xie L., Qian X., Ruan S., Zeng Q. (2020). Compositional dependence of pore structure, strengthand freezing-thawing resistance of metakaolin-based geopolymers. Materials.

[38] Pouhet R., Cyr M., Bucher R. (2019). Influence of the initial water content in flash calcined metakaolin-based geopolymer. Constr. Build. Mater..

[39] Chen L., Wang Z., Wang Y., Feng J. (2016). Preparation and properties of alkali activated metakaolin-based geopolymer. Materials.

[40] Barbosa V.F.F., MacKenzie K.J.D., Thaumaturgo C. (2000). Synthesis and characterisation of materials based on inorganic polymers of alumina and silica: Sodium polysialate polymers. Int. J. Inorg. Mater..

[41] Engineering ToolBox Thermal Conductivity of Selected Materials and Gases. https://www.engineeringtoolbox.com/thermal-conductivity-d_429.html.

[42] Kryvenko P., Kyrychok V., Guzii S. (2016). Influence of the ratio of oxides and temperature on the structure formation of alkaline hydro-aluminosilicates. East. Eur. J. Enterp. Technol..

[43] Hajimohammadi A., Ngo T., Kashani A. (2018). Glass waste versus sand as aggregates: The characteristics of the evolving geopolymer binders. J. Clean. Prod..

